# Stereotactic Radiosurgery and Stereotactic Radiotherapy for Malignant Skull Base Tumors

**DOI:** 10.7759/cureus.8401

**Published:** 2020-06-01

**Authors:** Yoshimasa Mori, Yoshihisa Kida, Yasuhiro Matsushita, Shinichiro Mizumatsu, Manabu Hatano

**Affiliations:** 1 Radiation Oncology and Neurological Surgery, Shin-Yurigaoka General Hospital, Kawasaki, JPN; 2 Radiology and Radiation Oncology, Aichi Medical University, Nagakute, JPN; 3 Neurological Surgery, Ookuma Hospital, Nagoya, JPN; 4 Neurological Surgery, Aoyama General Hospital, Toyokawa, JPN; 5 Neurological Surgery, Gamma Knife Center, Ookuma Hospital, Nagoya, JPN; 6 Cyberknife Center, Aoyama General Hospital, Toyokawa, JPN; 7 CyberKnife Center, Aoyama General Hospital, Toyokawa, JPN

**Keywords:** tumor, skull base, stereotactic radiotherapy, radiosurgery, chordoma, paraganglioma, meningioma, hemangiopericytoma, external auditory canal, nasal

## Abstract

The role of stereotactic radiosurgery/stereotactic radiotherapy (SRS/SRT) for malignant skull base tumors was summarized and discussed. The treatment of skull base tumors remains challenging. Their total resection is often difficult. SRS/SRT is one useful treatment option for residual or recurrent tumors after surgical resection in cases of primary skull base tumors. If skull base metastasis and skull base invasion are relatively localized, they can be candidates for SRS/SRT. Low rates of cervical lymph node involvement in early-stage (N0M0, no lymph node involvement or distant metastasis) nasal and paranasal carcinomas (NpNCa) and external auditory canal carcinomas (EACCa) have been reported in the literature. Such cases might be good candidates for SRS/SRT as the initial therapy. We previously reported the results of SRS/SRT for various malignant extra-axial skull base tumors. In addition, treatment results of early-stage head and neck carcinomas were summarized. Those of our data and those of other reported series were reviewed here to clarify the usefulness of SRS/SRT for malignant extra-axial skull base tumors.

## Introduction and background

Treatment of skull base tumors is challenging. Their total resection is often difficult, as they involve or are located adjacent to various important structures including cranial nerves, major vessels, and the brainstem [1]. Stereotactic radiosurgery/stereotactic radiotherapy (SRS/SRT) is one treatment option for residual or recurrent tumors after surgical resection in cases of primary skull base tumors such as chordoma, paraganglioma, and non-benign meningioma [2]. If skull base metastasis and skull base invasion are relatively localized, they are good candidates of SRS/SRT.

We have hitherto reported the results of SRS/SRT for various malignant extra-axial skull base tumors [3-13]. They were summarized with additional data on SRS/SRT of skull-base involvement of head and neck carcinomas. The role of SRS/SRT for skull base malignant tumors was summarized and discussed.

## Review

Various kinds of malignant tumors arise in the skull base (Table 1).

**Table 1:** Intracranial extra-axial malignant tumors including skull base tumors

Skull base tumors
i) Primary tumors
Chordoma/Chondrosarcoma, Paraganglioma, Meningioma (atypical/malignant), Hemangiopericytoma (Solitary fibrous tumor)
ii) Skull base cancers
a) Metastasis and invasion to the skull base
Distant metastasis from visceral cancers, Direct invasion of head & neck cancers
b) Early-stage head & neck cancers
Orbital, nasal, & paranasal cancers, External auditory canal cancers

In primary tumors, SRS/SRT is a treatment option after surgical resection with histological diagnosis. Localized distant metastases to the skull base could be treated on the basis of clinical diagnosis. Skull base invasion of head and neck cancers, most commonly recurrent after 1st line treatment including radiation therapy, can be treated by SRS/SRT, if the lesion is relatively localized. Furthermore, of various head and neck cancers, low rates of cervical lymph node involvement in early-stage nasal and paranasal carcinomas (NpNCa) and external auditory canal carcinomas (EACCa) have been reported in the literature [14-16, 17,18]. This might make them good candidates for SRS/SRT, instead of surgical extirpation.

Chordoma

Chordoma has a clinically malignant nature invading skull base structures. Total resection is always difficult, and marginal recurrence often develops. In such cases, SRS/SRT can be expected to control the recurrent or residual tumor instead of repeat surgery.

Mori et al. reported the treatment results of Gamma Knife SRS (GK-SRS) for chordoma in seven patients (Table 2) [3-6]. Although the sample size was small, the follow-up period was relatively long. Five-year local control rates (LCRs) of treated tumors, residual and recurrent after surgical resection, were 67%. With additional treatment for marginal and distant relapse, the five-year disease specific survival rate (DSSR) was 100%. They used mainly SRS by Gamma Knife (GK) (Elekta, Tokyo). In addition, SRT using Novalis (BrainLAB, Tokyo) was done for recurrence in one case, as the recurrent tumors were relatively large and fractionated SRT was deemed better to avoid adverse radiation effects. They delivered a single marginal dose of 15 to 20 Gy (median, 16 Gy) to the target tumors of 0.6 to 24 ml in volume. Temporary adverse effects were limited to only a single case in which right trigeminal nerve deficits, hypesthesia in the right cheek and right masseter muscle weakness were reported.

**Table 2:** Our published results of stereotactic radiosurgery for chordoma and paraganglioma *Relapse (local or distant) occurred in 13 (57%) **Relapse (local or distant) occurred in 4 (57%) ***One patient died of intracranial dissemination of the tumor at 87 mos. after SRS ****One patient died of tumor progression (91 mos.) and another died of lung carcinoma (17 mos.) *****One patient died of tumor progression (71 mos.) and another died of colon carcinoma (35 mos.) No. = number; pts. = patients; GK = Gamma Knife; SRS = Stereotactic radiosurgery; SRT = Stereotactic radiotherapy; yr = year; mos. = months; FU = Follow-up; LCR = Local control rate; Tx = Therapy.

Diagnosis	Author, Year	Modality	No. of pts. (No. of lesions)	Loca control	Survival	Additional Tx
Chordoma	Mori et al., 2014 [3]	GK-SRS	7 (7)	86% (3 yr) 67% (5 yr)	86%*** (FU, 68-175 mos.)	Repeat GK-SRS in four, Novalis SRT in one, Resection in one
Paraganglioma (Glomus tumor)	Kida et al., 2000 [4]	GK-SRS	4 (5)	100%	100% (FU, 24-72 mos.)	None
Non-benign meningioma (atypical / malignant)	Mori et al., 2013 [5]	GK-SRS	23 (26)	74% (1 yr) 52% (2 yr) 32% (3 yr)*	60%****	Repeat GK-SRS in four, Resection in six
Hemangiopericytoma (solitary fibrous tumor)	Tsugawa et al., 2014 [6]	GK-SRS	7 (10)	100% (1 yr) 92% (2 yr) 70% (3 yr)**	60%*****	Repeat GK-SRS in three

A number of papers have been published on chordoma SRS/SRT both with particle-beam and photon-beam [3, 19-23, 24-28]. However, in early-reported series the treatment results of both chordoma and chondrosarcoma were mixed [29]. Reported series dealing with only chordoma were summarized in Table 3 for particle-beams and in Table 4 for photon-beams [3, 19-23, 24-28]. Relatively larger volume cases were treated effectively by particle beam series with a more frequent fraction schedule.

**Table 3:** Reported results of particle beam radiation therapy for skull base chordoma *equivalent dose calculated with alpha/beta of 7 Pts. = patients; Vol. = volume; yr = year; fx. = fraction; P = proton; C = carbon; PTV = planning target volume; CGE = cobalt gray equivalent; RBE = relative biological effectiveness.

Author, Year	Modality	No. of Pts.	Median Vol. (ml)	Median Dose (Gy)	Local Control (%)	Survival (%)	Side Effects
Igaki et al., 2004 [19]	P	13	27.4 (3.3-88.4)	73.5 Gy / 33 fx. (64.0-103.1/20-38 fx.)*	46 (5 yr)	84.6 (3 yr), 63.6 (5 yr)	15%
Schulz-Ertner et al., 2007 [20]	C	96	80.3 (PTV[2 mm]) (13.9-594.2)	60 CGE / 20 fx. (60-70)	80.6 (3 yr), 70 (5 yr)	91.8 (3 yr), 88.5 (5 yr)	6%
Mizoe, 2016 [21]	C	33	32 (2-328)	60.8 GyE / 16 fx. (48.0-60.8)	85 (5 yr)	88 (5 yr)	3%
Uhl et al., 2014 [22]	C	155	70 (2-294)	60 Gy (RBE) / 20 fx. (14-15)	82 (3 yr), 72 (5 yr)	95 (3 yr), 85 (5 yr)	(not much)
Takagi et al., 2018 [23]	P or C	24 (13+11)	17 (0.4-131.1)	65.0 Gy (RBE) / 26 fx. (57.6-74.0/16-37 fx.)	85 (5 yr)	86 (5 yr)	50%

**Table 4:** Reported results of photon beam radiotherapy for chordoma *Only temporary adverse effects Pts. = patients; Vol. = volume; yr = year; GK = Gamma Knife; CK = CyberKnife; LINAC = linear accelerator-based system; SRS = stereotactic radiosurgery; ND = not described.

Author, Year	Modality	No. of Pts.	Median Vol. (ml)	Mean Margin Dose (Gy)	Local Control (%)	Survival (%)	Side Effects
Liu et al., 2008 [24]	GK	31	11.4 (mean) (0.47-27.6)	12.7 SRS (10-16)	64.2 (3 yr), 21.4 (5 yr)	90.9 (3 yr), 75.8 (5 yr)	none
Kano et al., 2011 [25]	GK	71	7.1 (0.9-109)	15.0 SRS (9-25)	66 (5 yr)	93.4 (3 yr), 80.2 (5 yr)	9%
Hafez et al., 2019 [26]	GK	12	7.0 (mean)	14.3 SRS	30 (3 yr), 25 (5 yr)	FU (12-120 mos.)	none
Mori et al. (ours), 2014 [3]	GK	7	5.1 (0.6-11.4)	16.9 SRS (15-20)	86 (3 yr), 67 (5 yr)	100 (5 yr), 50 (10 yr)	(14%)*
Zorlu et al., 2014 [27]	CK	11	14.7 (3.9-40.5)	30/5 fx. (median) (30-36/3-5 fx.)	80 (3 yr), 25 (5 yr)	91 (2 yr)	18%
Bugoci et al., 2013 [28]	LINAC	12	19.5 (8.5-73.2)	66.6/37 fx. (median) (48.6-68.4/27-38 fx.)	46.9 (2 yr), 37.5 (5 yr)	76.4 (5 yr)	ND

Photon beam seemed to be effective if the target tumors were not large and when large a single fraction dose was used.

After surgery or SRS/SRT timely assessment in cases developing local or marginal relapse with repeat SRS/SRT or surgical resection is indispensable to control chordoma, which shows a proclivity to develop repeat recurrence.

SRS/SRT is effective also in skull base chondrosarcoma residual and recurrent after surgical resection [30]. The prognosis was reported to be a little better for chondrosarcoma [31]. Murakami et al. reported a case of skull base mesenchymal chondrosarcoma, surviving more than 10 years after the initial surgery, with repeated SRS/SRT in addition to surgical resection 10 times [32]. They concluded that the extent of the initial resection is important for treatment outcome.

Paraganglioma (Glomus tumor)

Paraganglioma located at the skull base is also difficult to manage. Paraganglioma often arises around the jugular bulb system and involves cranial nerves such as the lower cranial nerves. It often exists over intracranial and infracranial spaces in a dumbbell shape. In addition, it is vascular-rich. Total resection of the tumor is often difficult.

Kida et al. reported the treatment results of GK-SRS for paraganglioma in four cases (five tumors) (Table 2) [4]. Five-year LCR of treated tumors residual and recurrent after surgical resection was 100%. They delivered a mean margin dose of 15.5 Gy (range, 15.0-16.0) to targets of 23.3 to 36.1 mm (mean 28.4) in diameter.

Recent reported series are summarized in Table 5 [4, 33-37].

**Table 5:** Reported results of stereotactic radiosurgery/stereotactic radiotherapy for paraganglioma (Glomus tumor) Pts. = patients; Vol. = volume; yr = year; mo = months; GK = Gamma Knife; LINAC = linear accelerator-based system; SRS = stereotactic radiosurgery; SRT = stereotactic radiotherapy; PFS = progression-free survival; FU = follow-up.

Author, Year	Modality	No. of Pts.	Median Vol. (ml)	Mean Margin Dose (Gy)	Local Control (%)	Survival (%)	Side Effects
Hafez et al., 2016 [33]	GK	22	7.3 (mean) (2.8-19.4)	14.7 SRS (12-16)	95.5 (5 yr, PFS), 95.5 (7 yr)	95.5 (5 yr), 95.5 (7 yr)	ND
Dobberpuhl et al., 2016 [34]	GK	12	up to 4.5 cm in diameter	(12-18)	100 (FU, 13-96 mo)	100 (FU, 13-96 mo)	none
Winford et al., 2017 [35]	GK	33	5.8 (mean) (0.9-11.4)	13.2 SRS (11-15)	95 (5 yr, PFS), 75 (10 yr)	100 (FU, 5.5-141 mo)	3.7%
Kida et al., (ours), 2000 [4]	GK	4	11.6 (7.5-23.5)	15.5 SRS (15-16)	100 (5 yr)	100 (5 yr)	none
Tripathi et al., 2019 [36]	GK SRS/SRT	10	29.9 (mean) (9.95-47.6)	(16-22 SRS, 22.4/2 fx. or 22.9/3 fx.)	100 (FU, 12-78 mo)	100 (FU, 12-78 mo)	1.8%
Gigliotti et al., 2018 [37]	LINAC SRS/SRT	16	11.7 (PTV)	(15 SRS, 21/3 fx., or 25-27.5/5 fx.)	88 (5 yr)	100 (FU, 55-87 mo)	6%

Tripathi et al. used a hypofractionated schedule using GK [36]. Gigliotti et al. reported the results of Linac-based SRT [37]. Recently, we treated two cases of large recurrent paraganglioma by two-volume staged GK-SRT (unpublished data).

Non-benign meningioma

Histologically 4-7% of meningiomas are considered atypical meningioma (AM) and 1-2% are malignant meningioma (MM) [5]. Non-benign meningiomas tend to recur within a relatively short term even after radical surgical resection.

Mori et al. reported the treatment results of GK-SRS for atypical and malignant meningioma in 23 cases (36 tumors) (Table 2) [5]. Three-year LCR in non-benign meningioma was 34%. Three-year DSSR was 91%. They delivered a mean marginal dose of 16.7 Gy (range 11.0 to 21.15) to the targets of 0.4 to 35.3 ml (median 8.6) in volume. They concluded that regular neuroradiological follow-up with timely identification of the development of new tumors or progression of the treated neoplasm followed by repeat management of SRS/SRT or microsurgery is important for prolongation of patient survival.

Acker et al. reported the results of CyberKnife SRS/SRT for AM and MM in 35 patients (127 tumors) [38]. Single-fraction SRS in the range of 15-18 Gy, or SRT of 21-24 Gy in three fractions, or 20-25 Gy in 4-5 fractions was delivered. The LCRs were 97%, 77%, and 67% at one, two, and five years, respectively, in AM and 66% each at one and two years in MM. The regional progression-free survival (PFS) was 93%, 73%, and 59% at one, three, and five years, respectively, in AM and 93% and 46% at one and two years in MM. The estimated distant tumor PFS in AM was 80%, 44%, and 44% at one, three, and five years, respectively, and 49% and 24% at one and two years, respectively, in MM. They treated the median planning target volume of 1.71 ml (range, 0.06 to 22.5) mostly by single-session SRS.

Recently Wu et al. published a review of the results of particle radiotherapy for WHO grade II (AM) and grade III meningiomas (MM) in 11 articles including 240 patients [39]. LCR at five years or at the end of the follow-up (median 145 months) ranged from 46.7 to 86%. Radiation necrosis was reported in 3.9% (3/77 patients) across the three studies available. Of them only one patient, who had had previous radiotherapy to the pituitary region, showed serious radiation necrosis (Common Terminology Criteria for Adverse Events (CTCAE) Grade 3).

Kaur et al. also published a review article on adjuvant radiotherapy for AM and MM [40]. The median five-year PFS and overall survival (OS) were 54.2% and 67.5%, respectively, for AM and 48% and 55.6% for MM. The complication rates were 11.1% for AM and 5.1% for MM. They described that incomplete resection and radiation dose < 50 Gy conferred significantly poorer five-year PFS. Adjuvant radiotherapy improved local control especially in patients with subtotally resected AM and MM, while inducing modest treatment toxicity. However, in patients with completely excised AM adjuvant radiotherapy did not confer any prognostic advantage, though in those with MM an advantage for adjuvant radiotherapy was indicated.

Hemangiopericytoma (Solitary fibrous tumor)

The clinical behavior of hemangiopericytoma is more aggressive than that of meningioma. This tumor is notable not only for its local aggressiveness and high rate of recurrence but also for its proclivity to develop extracranial metastases [6]. Spina et al. published a systematic review of 18 studies including 208 patients (366 tumors) [41]. Radiotherapy modality was GK in 14 series and CyberKnife (CK) or linear accelerator-based (LINAC) system in four. In the review of the literature, they described that the median time for recurrence was about five years, with PFSs of 96%, 49%, and 28% at one, five, and 10 years, respectively. In addition, the incidence of metastasis increased over time, amounting to 13%, 33%, and 64% at five, 10, and 15 years, respectively.

Tsugawa et al. reported the treatment results of GK-SRS for hemangiopericytoma in seven cases (Table 2) [6]. LCR was 92% and 70% respectively at three and five years. Three-year and five-year DSSR were both 100%. They gave a mean marginal dose of 16.5 Gy (range, 10 to 20) to the mean target volume of 4.1 ml (range, 0.3 to 23.9). Cohen-Inbar et al. reported a multicenter study using GK [42]. Eight centers had treated a total of 90 patients harboring 133 tumors. A median margin dose of 15 Gy (range, 2.8-24) was delivered to a median tumor volume of 4.9 ml (range, 0.2-42.4). Crude LCR at the last follow-up was 55% of tumors and 62.2% of patients. New remote intracranial tumors were noted in 27.8% of patients, and 24.4% of patients developed extracranial metastases. OS was 91.5%, 82.1%, and 73.9%, respectively at two years, four years, and six years. Local PFS was 81.7%, 66.3%, and 54.5%. They showed that the development of extracranial metastases influences OS, with PFS repeat (multiple) with SRS conferring additional benefit.

While hemangiopericytoma has a tendency to recur repeatedly, aggressive tumor management including repeat SRS/SRT or microsurgery for recurrent and newly developed tumors can prolong patient survival.

Distant metastasis and direct invasion of malignancies

Metastases to the skull base are challenging situations because they often involve critical structures including cranial nerves and induce progressive ipsilateral neurological deficits. Skull base infiltrations by direct invasion of a regional primary malignant tumor of head and neck cancers, for example along the cranial nerve routes, often occur. Both metastasis and invasion by carcinomas, when localized, can be treated effectively by SRS/SRT. A number of reported series have documented the effectiveness of SRS/SRT for both situations, which were mixed [43]. However, the respective results differed somewhat. Improvement of local cranial nerve impairment is more expected in cases of isolated distant metastases. Improvement of neurological symptoms was obtained in 91% (10/11) of cases of distant metastases in contrast to 40% (4/10) of direct skull base invasions by head and neck carcinomas (Table 6) [8,9]. Mori et al. delivered 30-50 Gy (mean 37.8) in 10-14 fractions to the planning target volume (PTV) of 8 to 112 ml (mean 61.6).

**Table 6:** Our reported results of stereotactic radiotherapy for distant metastases, direct invasion by malignancies, and early-stage head and neck cancers (nasal and paranasal cancers and external auditory canal cancer No. = number; pts. = patients; SRT = stereotactic radiotherapy; CK = CyberKnife; FU = follow-up; mos. = months; N0M0 = no lymph node involvement nor distant metastasis.

Diagnosis	Author, Year	Modality	No. of pts.	Loca control (crude)	Survival	Remark
Distant metastases from visceral cancers	Mori et al., 2010 [8]	Novalis-SRT	11	100%	36% (FU, 5-36 mos.)	Neurological improvement in 10 (91%)
Direct invasion by head & neck cancers	Mori, 2011 [9]	Novalis-SRT	15	69%	80% (FU, 2-12 mos.)	Neurological improvement in six (40%)
Nasal & paranasal cancers	Mori, 2005-2015 [9-13]	Novalis-SRT	4	100%	100% (FU, 6-34 mos.)	SRT was performed as the initial therapy for N0M0 cases
External auditory canal cancer	(unpublished data)	CK-SRT	4	75%	100% (FU, 24-72 mos.)	SRT was performed as the initial therapy for N0M0 cases

Minniti et al. also reported the effectiveness of SRT, good local control and neurological improvement, in cases of skull base metastases involving the anterior visual pathway [44]. They delivered 25 Gy in five fractions. In 51% of their patients clinical improvement of preexisting cranial nerve deficits was achieved.

Early-stage head & neck cancers

Of various head and neck cancers, it is reported that only 10-15% of early-stage nasal and paranasal carcinomas (NpNCa) have cervical lymph node (LN) involvement [14-16]. Also, patients with early-stage external auditory canal carcinomas (EACCa) rarely develop regional LN metastasis [17,18]. Such patients might be good candidates for SRS/SRT as an upfront therapy, instead of surgical extirpation, as the initial therapy. Crude control rates of early-stage NpNCa (four cases) and EACCa (four cases) after SRS/SRT were 100% (6-34 months) and 75% (19-166 months), respectively (Table 6) [9-13].

Indications and treatment planning, including optimal prescription dose, fraction schedule and field decision, will have to be established in future studies.

SRS and SRT

GK and cone collimated CK have similar dose distributions, steep dose fall-off around the target and relatively high doses inside the target. Gamma Knife procedures previously needed rigid head frame fixation with skull screws and were only suitable for single-session SRS. However, Icon (Elekta, Tokyo) with a relocatable thermoplastic facial mask system, good for fractionated SRT, has recently become available. We have used GK-SRS for relatively localized small tumors, especially postoperative residual and recurrent, in primary skull base tumors of chordoma, paraganglioma, meningioma, and hemangiopericytoma. If a relatively broad target with prophylactic surrounding area, i.e. the operative field of tumor bed, has to be included as the target, we have used Novalis or CK. Metastatic carcinomas with a highly invasive nature were targeted by LINAC, such as Novalis or TomoTherapy.

Considering chordoma, non-benign meningioma, and hemangiopericytoma, the feasibility of postoperative adjuvant therapy has to be established, even if the tumor is resected totally. The optimal treatment protocols for early-stage nasal and paranasal carcinomas and external ear canal carcinomas have to be established.

## Conclusions

SRS/SRT has been clinically applied for various malignant skull base tumors successfully. However, further investigation of larger populations with longer follow-up periods will be necessary to establish optimal indications and treatment planning considering the histological diagnosis, location, and individual circumstances, including whether it is to be the initial radiotherapy or retreatment of the tumors.

## Appendices

The authors have no financial or other conflicts of interest in relation to this research or its publication to declare.

The data on the EACCa patients were accumulated under the approval of the Ethical Committee Board of Shin-Yurigaoka General Hospital (20190520-2) and Aoyama General Hospital (19-02).
